# Conducting clinical trials in sub-Saharan Africa: challenges and lessons learned from the Malawi Cryptosporidium study

**DOI:** 10.1186/s13063-020-04620-8

**Published:** 2020-07-25

**Authors:** Neema Toto, Elaine Douglas, Markus Gmeiner, Lynn K. Barrett, Robert Lindblad, Lumbani Makhaza, Wilfred Nedi, Jacob Phulusa, Gerald V. Quinnan, Leigh A. Sawyer, Herbert Thole, Wesley C. Van Voorhis, Pui-Ying Iroh Tam

**Affiliations:** 1 Malawi-Liverpool Wellcome Trust Clinical Research Programme, Blantyre, Malawi; 2 Liverpool School of Tropical Medicine, Liverpool, UK; 3 Center for Emerging and Re-emerging Infectious Diseases (CERID), University of Washington, Seattle, WA USA; 4 Emmes, Rockville, MD USA

**Keywords:** Clinical trial, Developing country, Sub-Saharan Africa, Low-resource setting, Cryptosporidiosis, Diarrhea, Clofazimine

## Abstract

**Background:**

An effective drug to treat cryptosporidial diarrhea in HIV-infected individuals is a global health priority. Promising drugs need to be evaluated in endemic areas which may be challenged by both lack of resources and experience to conduct International Committee of Harmonisation-Good Clinical Practice (ICH-GCP)-compliant clinical trials.

**Methods:**

We present the challenges and lessons learned in implementing a phase 2A, randomized, double-blind, placebo-controlled trial of clofazimine, in treatment of cryptosporidiosis among HIV-infected adults at a single site in Malawi.

**Results:**

Primary challenges are grouped under study initiation, study population, study implementation, and cultural issues. The lessons learned primarily deal with regulatory system and operational barriers, and recommendations can be applied to other human experimental trials in low- and middle-income countries, specifically in sub-Saharan Africa.

**Conclusion:**

This study demonstrated that initiating and implementing human experimental trials in sub-Saharan Africa can be challenging. However, solutions exist and successful execution requires careful planning, ongoing evaluation, responsiveness to new developments, and oversight of all trial operations.

## Background

Clinical trials, particularly human experimental trials, are critical to learning the safety and efficacy of drugs and therefore to treating diseases [1, 2]. Trials are especially needed to address the high burden of disease in low- and middle-income countries (LMICs), specifically in sub-Saharan Africa. For as much as 50% of the global burden of disease, mostly due to infections, reside in sub-Saharan Africa [3].

Clinical trials testing drugs and vaccines that target specific diseases afflicting people in sub-Saharan Africa are logically best run in those countries. These trials can benefit from local healthcare knowledge and are better able to address context-specific questions that would then lead to more effective interventions [4]. Clinical research conducted in LMICs, particularly sub-Saharan Africa, build both research and health care capacity [4], which has been shown to strengthen health systems, expand health programming, and provide an evidence base for future health crises responses [5]. Major operational benefits of conducting trials in sub-Saharan Africa include the ease of recruiting trial participants and the availability of health workers with clinical research experience [4].

**Fig. 1:**
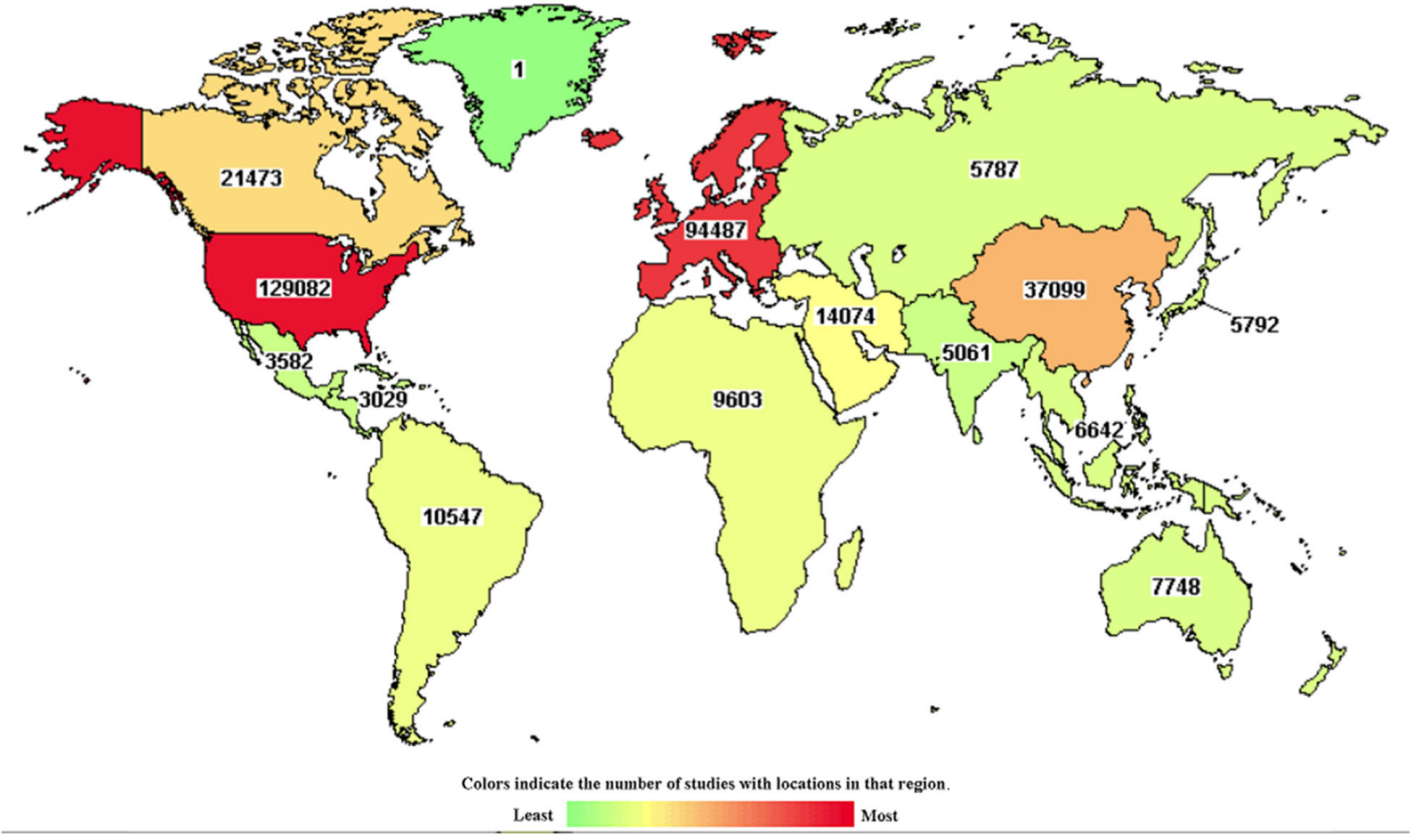
Distribution of clinical studies registered on ClinicalTrials.gov on April 2019 [6]

Despite these facts, only 20–30% of global clinical trials are conducted in LMICs and less than 10% in sub-Saharan Africa (Fig. 1) [1]. Several barriers to conducting research in sub-Saharan Africa contribute to this situation. These barriers include financial and human capacity, delays in regulatory and ethical reviews, complex logistical and financial systems, and competing demands [4].

In the developing world, *Cryptosporidium* infection and the associated diarrheal disease (cryptosporidiosis) is life-threatening in persons with HIV infection and in children younger than 5 years [7]*.* Currently, nitazoxanide is the only licensed drug for the treatment of cryptosporidiosis in immunocompetent persons after the first year of life [8].

The first human experimental trial to evaluate the therapeutic clofazimine (CFZ) for treatment of cryptosporidiosis was conducted in Malawi, a low-income country with limited experience conducting clinical trials and with even less experience conducting human experimental trials. This study was classified as a phase 2a trial evaluating the safety, tolerability, pharmacokinetics (PK), and efficacy of CFZ in cryptosporidiosis (Cryptofaz). It was a two-part study (A and B) that took place at Queen Elizabeth Central Hospital (QECH) in Blantyre, Malawi [9]. Part A was a randomized, placebo-controlled, double-blind clinical trial with a 1:1 ratio of intervention to control. Part B was an open-label study, comparing the PK of orally administered CFZ in HIV-infected individuals with and without *Cryptosporidium* diarrhea. Further details on the study protocol can be found elsewhere [9]. Recruitment began in December 2017 and was completed in January 2019.

Similar to the experience of other clinical trials conducted in sub-Saharan Africa, the Cryptofaz team encountered several logistical, operational, and implementation challenges [9]. Addressing these challenges effectively was key to conducting this clinical trial [10]. This article reports on the lessons learned. These lessons can be useful to other human experimental trials being considered or conducted in sub-Saharan African settings.

## Challenges

Topic areas are presented according to when they occurred during the clinical study. The challenges, lessons learned, and recommendations are listed in full in Table 1.

**Table 1:** Main challenges, lessons learned, and recommendations

Challenge	Lesson learned	Recommendations
**Study initiation**		
Contracts and regulatory approvals	• Contracts and agreements between multiple international institutions can cause delays. • Obtaining ethics and regulatory approvals in Malawi is a complex process that required significant time and effort from study staff.	• Begin the process of getting partner agreements even before the grant is approved. • Confirm commitment of subcontractors during the grant process. • Ensure the site has dedicated in-country person experienced with the ethics and regulatory agencies to focus on the processes and check outcomes. In particular, they should: ° Facilitate study team’s finalization of protocols and other documents to keep them in line with submission dates. ° Check on progress immediately after review deadline. ° Ensure that approval documents are received. ° Check returned documents for accuracy.
Trial insurance	• Obtaining trial insurance in Malawi is complicated. • The sponsor had to work through an institutionally sanctioned insurance broker. • The broker contracted with a multinational insurance company who then contracted with a local insurance firm in Malawi. • Additionally, a local insurance firm then had to front the policy before it could be endorsed by the National Commission for Science and Technology (NCST).	• Sponsor and study team should coordinate with each other and with the various companies in order to keep the process moving and ensure that requirements are met.
Staffing	• It takes planning, creativity, and tenacity to recruit and retain excellent study personnel. • Experienced personnel were generally already under a contract either with a different MLW clinical trial or other research institutions in the country. • When promising clinicians (without specific clinical trial experience) are hired, training, oversight, mentorship, and timely feedback can ensure that staff succeed.	• Plan hiring early. • Contact investigators whose studies are ending. • Whenever possible, draw from study teams that have implemented similar studies. • When promising staff are hired: ° Provide basic theoretical training on clinical trials/clinical research. ° Provide specific hands-on training. ° Ensure ongoing support/supervision from experienced local and partner lab and clinical personnel.
**Study population**		
Information about study population	• Adequate information about the study population is key. • The number of potential subjects that would meet inclusion criteria and fail due to exclusion criteria was unknown, e.g., an exclusion criteria of potassium < 3.5 mEq/L was almost universal in this population with prolonged diarrhea.	• Conduct pilot study specific to the planned trial in order to gain a clear understanding of the realities. • Focus especially on data that relates to inclusion and exclusion criteria. • For correctable inclusion criteria, such as potassium levels, consider correction and a needed retesting value.
Slow enrollment rate	• Expected enrollment rate was based on a preliminary study that did not match study requirements. • Climate change is affecting weather patterns; this impacts the prevalence of pathogens. • Oral potassium supplements can address hypokalemia within the recruitment time period, thus allowing subject eligibility. • Chest X-rays and sputum GeneXpert alone are not reliable in detecting TB; urine LAM identifies undiagnosed TB in immunocompromised HIV-infected patients. • Hospital-based recruitment was insufficient; expanding to outreach sites led to an increase in the number of potential subjects approached and subjects enrolled.	• Conduct pilot study in line with study parameters, particularly related to study population. • Verify the climate conditions that existed when baseline data re: recruitment rate was gathered. • Plan for changing weather patterns, i.e., conduct the trial in multiple sites simultaneously. • Be more conservative about estimates when preliminary study inclusion/exclusion criteria do not match study criteria. • Identify strategies for dealing with slower than expected enrollment. • Consider setting study enrollment target off-ramps.
Study population health status	• Potential participants were severely immunocompromised and had multiple opportunistic infections. • Many were failing on ARV treatment. • Related to above, many subjects were found to have undiagnosed TB early in the study. • Local clinical staff are able to ensure that subjects can access available and appropriate treatment.	• Ensure consultation with expert clinicians. • Consider ramifications of potential participants failing first-line ARV treatment, thus eligible for second-line treatments (identified in exclusion criteria) rendering them ineligible. • Plan for extensive screening procedures (such as urine LAM to rule out extrapulmonary TB) to isolate exclusionary conditions. • Facilitate referral for care and management. • Provide clear instructions at discharge related to worsening conditions and follow-up with subjects.
High mortality rate	• Subjects with diarrhea, HIV, and *Cryptosporidium* had CD4 counts uniformly under 100 and had multiple underlying conditions that contributed to the mortality rate. • Mortality rate of 20% in part A was higher than the anticipated rate of 15% (projected from published data re: HIV+ individuals with diarrhea). • Experienced HIV clinicians at the Data and Safety Monitoring Board (DSMB) concluded that mortality rate seen in this study was not unexpected.	• Before study begins, get input from experienced in-country clinicians about expected mortality rates for the specific population. • Ensure that clinicians refer very ill subjects to appropriate clinics to ensure proper care and management.
**Study implementation**		
Lab equipment	• Identification of suppliers and acquisition of critical lab equipment and supplies took more time than anticipated. • Maintenance of one malfunctioning piece of equipment not easily obtainable in Malawi (in this case, the Thermal Cycler, polymerase chain reaction [PCR] machine) caused significant delays.	• Study team, including partners, should collaborate to identify suppliers well ahead of trial initiation. Establish realistic delivery timelines. • Develop close communication with suppliers to emphasize the critical importance of equipment to the study to ensure equipment is delivered in a timely manner and maintained. • Maintain close contact with technical personnel from suppliers to facilitate resolution of malfunctioning equipment. • Identify backup labs early to use when lab machine is faulty, or reagents have run out.
Lab testing	• Study required new skill sets for site staff, particularly lab staff. • Viability of ultra-cold cell specimens is not guaranteed. • Clinical lab at site hospital did not run samples quickly and not at night nor over weekends, causing unanticipated delays. • PK sample collection and other procedures spaced too closely together can lead to errors. • Enrollment rate of 2 subjects per week allowed adequate time for processing of lab tests.	• Provide expert trainers to work with the site lab personnel to establish new complicated techniques and maintain feedback for ongoing troubleshooting. • Consider sending lab personnel to partner labs to observe routine processes before initiating the trial. • Develop a backup plan for cell culture including backup shipments and alternative substrates. • Ensure adequate and ongoing supply of reagents. • Make arrangements to do clinical labs via study labs or contract with the clinical lab to run the samples immediately. • Consider rolling admission days to ensure adequate time for PK and other studies.
Randomization timing	• Some procedures required study staff with regular weekday hours to come in on holidays and weekends.	• Ensure that protocol is in line with work schedules to ensure that adequate staffing is available.
Protocol amendments	• Getting protocol amendments in place (and adjusting related study documents) took more effort and time than anticipated. • Protocol revisions impact all downstream data entry and document revisions. • This can slow the progress of the study.	• Anticipate protocol amendments when conducting studies in new areas. • Plan for the impact of amendments and ensure that study deadlines can be reached. •Try to ensure quick consensus on proposed protocol amendments by all concerned staff and partners.
Data collection forms (DCFs) and data entry	• DCFs contained unclear or incorrect fields. • Missing data fields and data entry errors early in the study caused numerous data queries. • Significant staff time was required to correct the forms and resolve the queries.	• Perform mock run-throughs of completing DCFs and data entry with the clinical and data entry staff at site initiation visit. • Site data entry staff perform ongoing audits of data fields before submitting. • Ensure continuous and open communication among the clinical, data entry, and CRO staff to discuss queries and related data issues.
Physical space	• Lack of space at QECH prevented separate clinic rooms for subjects. • Part B (non-diarrheic, non-*Cryptosporidium-*infected individuals) needed separate inpatient space to prevent exposure to *Cryptosporidium* oocysts. • If study had recruited the expected number of subjects, trial participants could have taken beds needed for non-study participants. • QECH infrastructure had to be upgraded to create office space for the clinical staff.	• Analyze space availability at study site against study space needed (including office space). • Ensure that adequate space is available to support good health outcomes for both study participants and non-study participants.
Site Internet connectivity	• Internet connection is critical when an electronic data system is used.	• When electronic systems are key to study implementation, ensure the study site has a strong Internet connection and identify backup plans, several if possible. • Consider cellular data as a way to ensure nearly continuous connectivity.
**Cultural issues**		
Blood draws	• Superstition can cause subjects, their families, and communities to object to study procedures. • Uninformed ward staff can perpetuate misinformation about study-related procedures. • Staff awareness of community perceptions and potential threats to the study is extremely useful to prevent problems.	• Ensure strong links to District Health Offices. • Suspend community recruitment during volatile periods. • Provide ongoing community, subject, and ward staff education on the need for frequent blood draws and the small amount of blood being taken. • Ensure proper consenting with subjects and guardians. • Draw blood in a separate room, away from the ward.
Food supplement palatability	• Some subjects disliked the selected food supplement, and one could not eat it. • Mixing food supplement with instant maize porridge (a common food staple) improved subjects’ ability to consume it.	• Perform quick assessment before study initiation to ensure the target population can consume supplement. • Adjust supplement, if needed, to make it more suitable.

### Study initiation

#### Contracts and regulatory approvals

Agreements between key international institutions took more time (several months when there was a need to accomplish this within several weeks) than anticipated. Similar to the experience of other clinical trials being conducted in developing countries, obtaining ethics and regulatory approvals in Malawi required significant time and effort from study staff [4].

#### Trial insurance

In order to get the required trial insurance in Malawi, the sponsor and local site had to contract with four insurance companies. We were required to work through an institutionally sanctioned insurance broker and this created complications. The multiple layers of bureaucracy involved in getting the policy approved increased the time to trial commencement.

#### Staffing

Hiring expert clinical trial personnel was a lengthy process. Identifying seasoned candidates with clinical trial experience is especially challenging in a less-developed setting. Local populations generally have limited experience conducting clinical trials—though this is increasing.

### Study population

The study population involved HIV-infected adults with three or more days of diarrhea who met all the inclusion and none of the exclusion criteria [10].

#### Slow enrollment

Initial enrollment projections (66 subjects enrolled in 9 months) were based on a preliminary study previously conducted in Blantyre, Malawi, where a rapid diagnostic test was used on patients hospitalized with diarrhea over a 3-month period to obtain rough estimates of the prevalence of *Cryptosporidium* in the hospitalized population (17%). The preliminary study captured information on HIV status and clinical stage, diarrhea duration, and age, but not information on the laboratory and clinical exclusion criteria, such as electrolyte abnormalities or tuberculosis testing. Unfortunately, the actual enrollment rate for the Cryptofaz study (15 subjects in the first 10 months) did not match the projections.

There are several factors that accounted for the slow enrollment. Initial recruitment was based on a diarrhea duration of > 14 days, and it was difficult to find subjects with this diarrhea duration who were also *Cryptosporidium* positive by PCR. Therefore, the study team modified the inclusion criteria in an effort to increase enrollment. The required diarrhea duration was shortened as well as the duration an individual could be on antiretroviral therapy (ART). In addition, outreach was expanded to include referrals from health clinics in the surrounding urban Blantyre area. Furthermore, an unusually dry season during the recruitment period appeared to play a role in the low infection rate in the Blantyre population.

However, even with these study modifications, the enrollment rate did not increase to the anticipated level. Ultimately, the study team determined that this enrollment rate was too slow to be feasible, and the study was stopped due to an insufficient recruitment rate after enrolling 20 subjects in part A of the trial (instead of the anticipated 56 subjects). Correspondingly, the interim analysis, planned at 20 subjects, became the final analysis.

#### Health status

The design of Cryptofaz was based on a pilot study that did not include information on the health status of potential subjects. Therefore, we were not aware of potential laboratory abnormalities and occult disease until screening. The study identified multiple potential subjects who were failing their antiretroviral treatment (ART) despite being on treatment for months or years. Related to the low CD4 counts, many subjects were found to have undiagnosed TB. Also, many potential subjects were found to be severely hypokalemic because of their diarrhea and had to receive potassium supplementation prior to enrollment. The study provided care and close clinical follow-up to these subjects.

#### High mortality rate

In this resource-limited setting, the team found that many subjects who fit the inclusion and exclusion criteria were extremely ill and some did not survive the study period. Hence, the mortality rate (20%) was slightly higher than initially estimated (15%). With the addition of LAM TB screening, enrollment was affected (decreased) as more cases of undiagnosed TB were detected and the mortality rate also decreased.

### Study implementation

#### Lab equipment

The study required acquisition and installation of critical lab equipment. Most of this equipment had to be procured from outside Malawi. Therefore, lengthy shipping times had to be worked into the study timeframe.

#### Lab testing

Commonly, sites new to clinical research will require new skill sets. Cryptofaz was a technically complex study with multiple new laboratory tests and procedures. For example, examination for reliability of qPCR data was a new procedure for the study team, and the team required ongoing review, feedback, and troubleshooting support from one of the study’s partner institutions.

#### Randomization timing

The original protocol required randomization to occur simultaneously with study investigational product, preparation, and dispensation. This required both the pharmacist and data manager to be available nights and weekends, times outside of these staff normal working hours. Therefore, staffing schedules were modified to accommodate any potential out-of-hours study-related work needed.

#### Protocol amendments

Cryptofaz was an experimental study that had not been performed before. Thus, the characteristics of the potential participants and the enrollment rate were unknown. For these reasons, the trial required four protocol amendments to optimize the clinical trial recruitment, enrollment, and completion.

#### Data collection forms and data entry

While the clinical team reviewed each data collection form (DCF) and a data entry plan was developed before study implementation, ensuring an accurate data entry process required substantial effort and subsequent cleaning up of the data after entry.

#### Physical space

The study required dedicated space for inpatient treatment as well as for administration of the study. At the time of study initiation, QECH did not have available space to dedicate to the study.

#### Site Internet connectivity

The study relied on an electronic data system whereby data was to be transferred to the Contract Research Organization (CRO) in a timely manner for oversight and monitoring. The local site Internet was unreliable, and this slowed down training and data entry.

### Cultural issues

#### Blood draws

Cryptofaz required blood draws for the PK studies. In Malawi, as in many sub-Saharan African countries, collecting blood comes with challenges. This relates to the conception of blood as a “life force” (thus strength is drained by losing blood) and to rumors of “blood suckers” (vampires) who come at night and suck people’s blood, thereby removing power and fertility from the person [11].

A few months before the study was to begin, groups of people in and near Blantyre attacked several persons (including health care workers) that were accused of being “blood suckers.” During study implementation, these fears threatened to interfere with the study. During a regular blood draw of a subject, her guardian became agitated about the number of tubes and accused the clinician of being a “blood sucker.” Others in the ward joined in with the accusations. Extensive sensitization and counseling of both ward staff and guardians were required to improve expectations of study procedures.

#### Food supplement palatability

The Cryptofaz study required nutritional consistency, both in terms of content and timing of meals. However, as nutritional consistency and quality of meals provided by QECH or guardians could not be ensured, the study team identified “Plumpy’Nut” [12] and “Plumpy’Soy” [13] as alternative appropriate food supplements and found a local source for these products. Some subjects found the supplement to be unpalatable, and therefore, the team had to identify ways to make the taste more acceptable.

## Conclusion

The Malawian Cryptofaz study is an example of a successful human experimental trial conducted in a low-income country. Barriers to the conduct of clinical trials in developing countries have been systematically reviewed and fall under five unifying themes: lack of financial and human capacity, ethical and regulatory system obstacles, lack of research environment, operational barriers, and competing demands [2]. However, few of the clinical trials listed in the reviews were from Africa and even fewer from the “least developed” countries as designated by the Development Assistance Committee (DAC) [14].

Sub-Saharan African countries face enormous challenges in conducting high-quality clinical trials. However, in the case of the Cryptofaz trial in Malawi, the study team encountered challenges, particularly in the area of regulatory system and operational barriers, but were able to overcome most. As a result, the capacity at the investigational site for conducting clinical trials was built and/or strengthened. This was particularly true in relation to the site’s capabilities in clinical services, data management, pharmacy, study coordination, governance, and human resources. The challenges, lessons, and recommendations from Cryptofaz are specific to the study’s context, but may prove useful to other human experimental trials in sub-Saharan Africa.

## Data Availability

The datasets used and/or analyzed during the current study are available from the corresponding author on reasonable request.
